# Fine-tuning protein embeddings for functional similarity evaluation

**DOI:** 10.1093/bioinformatics/btae445

**Published:** 2024-07-10

**Authors:** Andrew Dickson, Mohammad R K Mofrad

**Affiliations:** Departments of Bioengineering and Mechanical Engineering, Molecular Cell Biomechanics Laboratory, University of California, Berkeley, CA 94720, United States; Departments of Bioengineering and Mechanical Engineering, Molecular Cell Biomechanics Laboratory, University of California, Berkeley, CA 94720, United States

## Abstract

**Motivation:**

Proteins with unknown function are frequently compared to better characterized relatives, either using sequence similarity, or recently through similarity in a learned embedding space. Through comparison, protein sequence embeddings allow for interpretable and accurate annotation of proteins, as well as for downstream tasks such as clustering for unsupervised discovery of protein families. However, it is unclear whether embeddings can be deliberately designed to improve their use in these downstream tasks.

**Results:**

We find that for functional annotation of proteins, as represented by Gene Ontology (GO) terms, direct fine-tuning of language models on a simple classification loss has an immediate positive impact on protein embedding quality. Fine-tuned embeddings show stronger performance as representations for K-nearest neighbor classifiers, reaching stronger performance for GO annotation than even directly comparable fine-tuned classifiers, while maintaining interpretability through protein similarity comparisons. They also maintain their quality in related tasks, such as rediscovering protein families with clustering.

**Availability and implementation:**

github.com/mofradlab/go_metric

## 1 Introduction

Despite decades of research, protein function is still only sparsely understood, with a huge percentage of proteins uncharacterized, or incompletely understood. However, the sequence information on amino acids making up proteins is typically known with high accuracy, meaning that any effective method for predicting functions from sequence would fill an enormous gap in biological research.

Datasets such as the Gene Ontology (GO) describe thousands of possible functions for proteins in a comprehensive hierarchy, which works as a rigorous target for machine learning prediction (Ashburner *et al*. 2000). Proteins may be annotated with one or more GO terms independently, making functional annotation a multi-class, multi-label problem setting. GO annotation is characterized by an extremely high number of classes, but also by relative rarity of most classes. While the most frequent annotations are attached to most of the protein dataset, the vast majority occur only for a handful of samples. Out of the top 800 terms used for our experimental dataset, half of the terms occur in <0.2% of datapoints.

Heavy effort is put to predicting GO annotations for proteins, with trends toward fine-tuned language models as the strongest modern classifiers. However, the most popular methods for GO annotation in practice are based on sequence similarity, like BLAST (McGinnis and Madden 2004, Conesa *et al.* 2005). Despite lower accuracy, they have the advantage of explaining their predictions with examples of similar proteins with known function.

A simple way to reproduce this functionality with deep learning models is to make annotation predictions indirectly, by embedding proteins in some high-dimensional latent space and then classifying them with K-nearest neighbors using Euclidean or Cosine distance as a similarity metric, an approach with precedents in text search (Schütze *et al.* 2022). Embedding distances have already been applied toward protein homology prediction (Muennighoff 2022), and directly derived from BERT representations in tasks such as drug interaction prediction (Djeddi *et al.* 2023). Here, we investigate the utility of this approach toward protein annotation, in comparison with BLAST and with standard deep learning models, and demonstrate that it is highly effective under typical fine-tuning procedures. It joins a growing number of uses for self-supervised language models in modern proteomics (Cui *et al.* 2023, Lin *et al.* 2023, Madani *et al.* 2023, Theodoris *et al.* 2023, Wang *et al.* 2023).

## 2 Materials and methods

### 2.1 GOBench dataset

For this problem setting, we derive an annotation dataset from existing databases. All experiments are run on a custom built GO dataset from our previously developed gobench.org web application, with human-reviewed experimental codes (Rogers and Ben-Hur 2009) selected and otherwise default settings (Dickson *et al.* 2023).

To limit the effect of missing annotations, our dataset settings constrain our study to the set of human-reviewed proteins in SwissProt (Consortium 2020) and we only train or evaluate on proteins with at least one annotation. Additionally, because annotation performance is dominated by performance on the most common GO terms, we limit training to GO terms which occur over 50 times in our database. The final dataset generated by the gobench.org web application is based on the Gene Ontology Annotation dataset (Huntley *et al*. 2015), with similar methodology to the CAFA competition (Zhou *et al*. 2019). In total, it contains 112 000 proteins, with an average of 10 annotations for each protein.

The dataset contains 112 000 unique, human-reviewed proteins, with an average of 10 associated annotations out of 865 allowed possibilities. To avoid contamination of the testing set, proteins are clustered into related families before partitioning into training/testing, again following the base settings of the gobench application.

### 2.2 Language model fine-tuning for gene ontology annotation

Fine-tuning is now a well-known strategy for boosting model performance in low-data regimes (Reimers and Gurevych 2019). We use two pretrained language models for testing, both trained based on BERT methodology. The Rostlab ProtBERTBFD model is a transformer model pre-trained to predict masked amino acids in protein sequences from the BFD dataset (Steinegger and Söding 2018), while the ESM2 model is similarly pretrained on 250 million sequences retrieved from UniParc (Consortium 2020, Lin *et al.* 2023).

Both BERT models use a sequence of tokens representing protein residues as input, with a CLS token prepended to represent global information. BERT models are trained to predict masked sequences (Devlin *et al.* 2019), so the output is a sequence of embeddings, with one embedding for each input token. However, our problem setting of GO classification requires global predictions for the entire protein. Following fine-tuning convention, we average protein residue embeddings and add them to the model’s CLS embedding to get a single, 1024 dimensional, representation. We then linearly transform the embedding into 865 logit values, with one logit prediction for each GO class. This allows large language models (LLMs) to function as a multi-class, multi-label classifier for GO terms, with some extra parameters from our final linear transform. During fine-tuning, classifier LLMs are trained to minimize binary cross-entropy loss on GO class labels until convergence. We directly use the internal model embedding prior to the final classification layer as a protein representation.

### 2.3 BLAST annotation

We use BLAST as a strong baseline method for protein classification, based on the DiamondBlast method from DeepGOPlus (Kulmanov and Hoehndorf 2019, Buchfink *et al.* 2021).

DiamondBLAST is a faster variant of BLAST, which returns bitscores indicating similarity to each protein in a database, for each query protein in our testing dataset.

For a query protein $P_{q}$ the score of a GO class *g* is an average of every associated database protein returned by DiamondBLAST, weighted by bitscore. Queries are run using default settings, which provide strong classifier performance in practice.

$$s(P_{q},g)=\frac{\sum_{P_{B}}bi\mathrm{tscore}(P_{Q},P_{B})I(P_{B}\;\mathrm{has}\;g)}{\sum_{P_{B}}bi\mathrm{tscore}(P_{Q},P_{B})}$$

### 2.4 Baseline deep learning classifiers

We train deep learning classifiers from scratch as a point of comparison against fine-tuning large language models or BLAST. Deep convolutional models which aggregate large filters are known to be a strong alternative (Kulmanov and Hoehndorf 2019), so we select them as our predominant strategy, and use a reimplementation of the DeepGOPlus architecture to generate GO class predictions. As with our language models, we use internal model embeddings as protein representations.

### 2.5 Baseline optimization

Baseline machine learning models are trained until convergence, using the pytorch and the pytorch-lightning frameworks (Falcon and The PyTorch Lightning team 2019, Paszke *et al.* 2019).

DeepConv models require many important hyperparameters to be chosen, including learning rate, number of convolution kernels, maximum kernel size, and final embedding size, which may be very different for our particular GO dataset. We follow a Bayesian optimization process for these four hyperparameters, and select our final values to maximize F1-score on our dataset after 80 trial training runs through Optuna (Akiba *et al.* 2019).

### 2.6 Multilabel KNN from protein embeddings

Protein sequences are converted to high-dimensional embeddings with one of our pre-trained, fine-tuned, or convolutional language models. Embedding size is 1024 for our pre-trained and tuned models, and 128 or 2048 dimensional for the convolutional models.

For KNN classification, all proteins in training and evaluation datasets are first embedded by some model. The training embeddings are treated as a reference database, with each protein embedding associated to a positive or negative assignment for each GO class based on label data.

Metric distance is defined as the Euclidean distance between vectors. For each evaluation protein, we search for the K training embeddings with the smallest distance. Then for each GO class, we estimate the class probability by the weighted percentage of nearest-K training embeddings positively annotated with this class. We evaluate KNN performance across a range of K for all models, and find that performance is relatively robust to the specific value of *K*, but maximized at roughly K=5, which we adopt for all model experiments. Weighting neighbors by inverse distance slightly improves performance and robustness to K, so distance weighting is also included in final evaluations. We find that Euclidean distance achieves higher F1 score than cosine for all models, and for pre-trained models in particular, and adopt Euclidean distance for all subsequent KNN experiments (Supplementary Fig. S1).

### 2.7 Machine learning codebase

All experiments are run through python scripts on in-house servers, with machine learning training executed on a NVIDIA RTX A6000 GPU, using pytorch and pytorch-lightning frameworks. All code is included in the github.com/mofradlab/go_metric repository.

## 3 Results

### 3.1 Fine-Tuning attains highest performance for GO annotation

As a reference baseline, we reproduce a subset of existing work in deep learning, fine-tuning pretrained models, and annotation through sequence comparison. We first use a modified version of BLAST based on prior work from DeepGOPlus (Kulmanov and Hoehndorf 2019) as a strong baseline. In practice, we find that it’s significantly stronger than typical BLAST approaches because it estimates protein annotation probabilities with a weighted average of similar proteins, instead of a direct comparison to the most similar protein.

We train convolutional models based on prior work in deep protein annotation (Kulmanov and Hoehndorf 2019, Sandaruwan and Wannige 2021), and extensively hyperparameter tune them to maximize their strength as a baseline.

**Figure 1.**
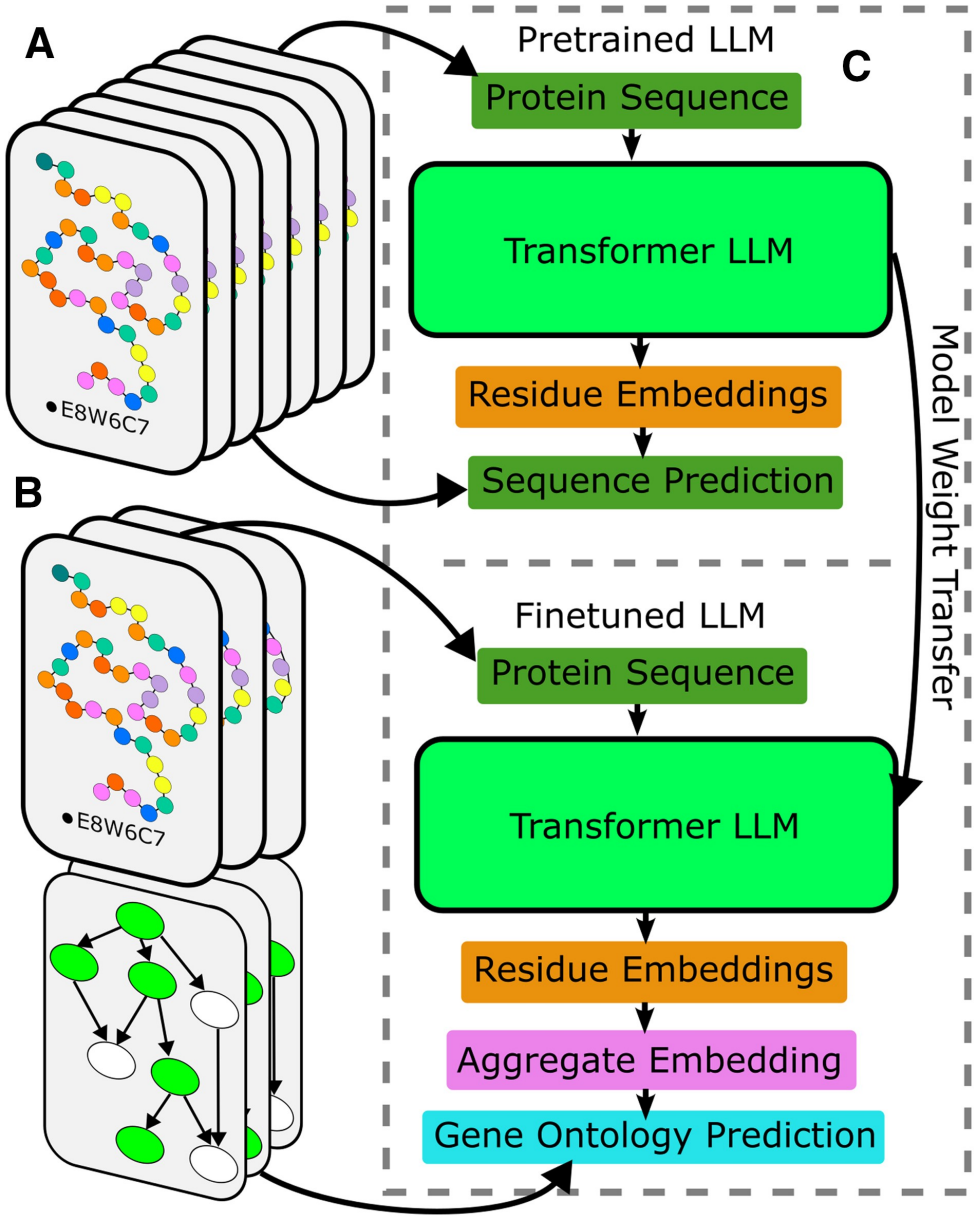
(A) Dataset structure for self-supervised language models. Pre-trained LLM’s are built to predict randomly masked residues of known protein sequences, with no auxilary data. Because of the simplicity of the dataset, it is much larger (3.83 hundred billion tokens) (Elnaggar *et al*., 2022). (B) Dataset structure for fine-tuning on protein annotations. Dataset is built from protein sequences paired with a set of Gene Ontology annotations, organized hierarchically. The fine-tuning dataset is relatively small, with 112 000 proteins. (C) Deep-learning model architectures used for evaluation. The pre-trained LLM predicts protein sequences with the transformer architecture by predicting an embedding vector for each protein residue and putting each residue through a classifier head. Weights and architecture from the pre-trained model are transferred to an annotation model for fine-tuning. Here, LLM models are modified to average the residue embeddings and add them to the BERT CLS token embedding to form a sequence level representation, which is then fed into a classifier layer.

We also finetune pre-trained LLMs for classification. For comparison, we finetune the Rostlab BERT-BFD (Elnaggar *et al.* 2022) and 650M parameter ESM model (Lin *et al.* 2023). Both are initially trained to uncorrupt masked residues in protein sequence, following the BERT architecture (Devlin *et al.* 2019). We convert to a classifier by averaging final, internal sequence embeddings into a single vector, and passing them through a task-specific, linear classifier head, following the example of previously successful finetuning strategies (Reimers and Gurevych 2019). With the exception of the linear head, all model weights in the annotation model are copied from the original pre-trained version of BERT. All model weights are then fine-tuned for GO annotation prediction on a slanted triangular learning schedule until convergence (Howard and Ruder 2018). Weight transfer, and pre-training and fine-tuning datasets are depicted in Fig. 1.

The average residue embedding vector is directly useful as a protein representation, and has already been used to make GO annotations using K-nearest neighbors in embedding space (Littmann *et al.* 2021). We evaluate the effect of fine-tuning by comparing the pre-trained model embeddings with fine-tuned versions taken directly from the classifier.

Following the example of CAFA (Zhou *et al*. 2019), all models are evaluated with precision-recall and MIS-RUS curves. Performance is summarized in the form of F-max and S-min metrics which give the F1 and S scores when model logit predictions are thresholded with the optimum value. Both are described in detail in an early CAFA paper (Radivojac *et al.* 2013), with S-min putting higher emphasis on rare GO classes (Clark and Radivojac 2013). Because missing data is a significant problem for GO datasets (Gillis and Pavlidis 2013, Gaudet and Dessimoz 2017), we take the extra step of calculating an ‘F-Estimate’ score, which is an estimate proportional to the true F1 score with no false negative labels (Bekker and Davis 2020). Relative F-estimate scores match F-max scores in nearly all cases, indicating that our testing dataset is a good proxy for real world performance.

**Figure 2.**
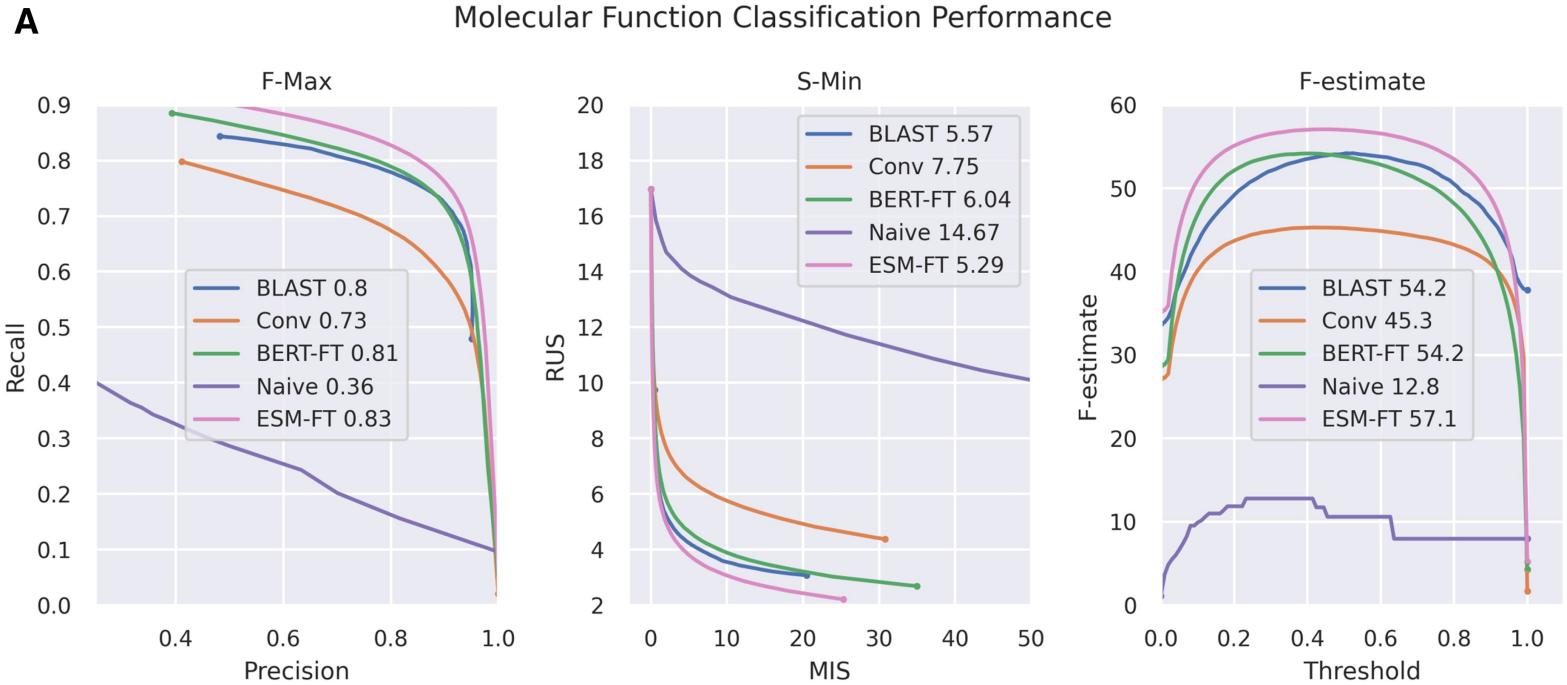
(A) Performance curves for each of our initial classifier models. Curves for naive class prediction based only on prior probabilities of GO classes are included, but because their performance is universally poor, they are excluded from further figures. Precision-recall and F-estimate curves show fine-tuned models as strictly stronger than alternatives, including BLAST, while MIS-RUS curves show BLAST as stronger than the fine-tuned BERT-BFD model at certain thresholds.

All loss curves are depicted in Fig. 2, showing relative model performance for the generic GO annotation task. Model results show that BLAST is extremely competitive, matching previous results for GO prediction datasets (Kulmanov and Hoehndorf 2019, Zhou *et al*. 2019).

Our major takeaway is that the only classifier models that directly surpass sequence comparison are fine-tuned language models with a classifier head. Both BERT-BFD and ESM improve over BLAST for both F-max and F-estimate scores, and fine-tuned ESM remains stronger for S-min scores. Although similarity search with pre-trained embeddings is highly desirable for interpretable results, it is initially weaker than directly training a classifier.

### 3.2 Fine-tuned KNN models outperform direct classifiers

**Figure 3.**
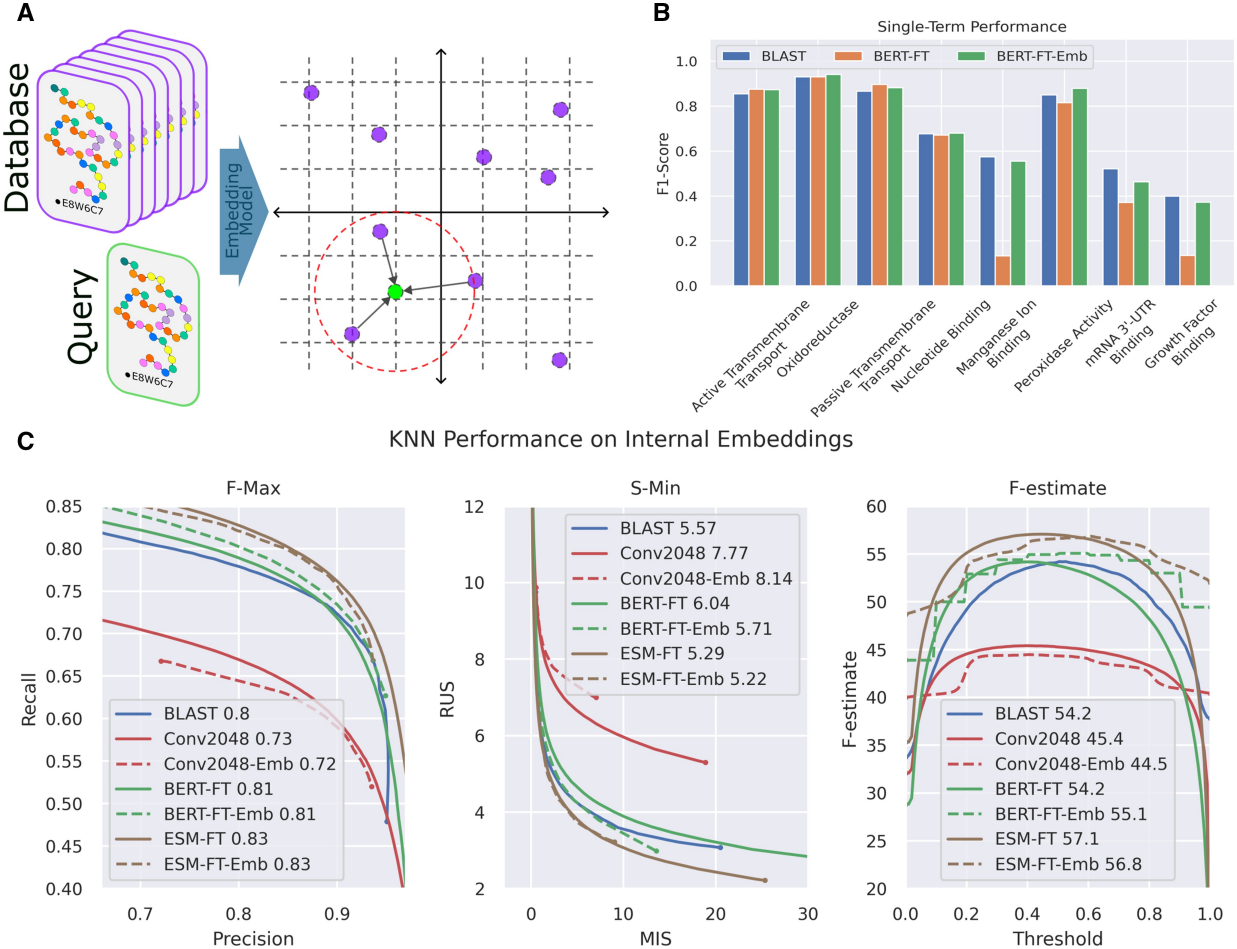
(A) Deep K-Nearest Neighbors scheme. Proteins in training data are embedded using either a large language model or a convolutional model in our results. Proteins in the validation or testing dataset are then embedded by the same model, so that their closest neighbors from the training database can be queried. Annotation probability is then calculated as the average frequency of GO classes within nearest neighborhood. (B) Model F1 scores on selected specific GO classes, at *P* = .5 threshold. (C) Performance curves for classifiers relative to KNN for their internal embeddings. BLAST and pre-trained language model curves included for reference. Classifier models indicated with solid lines, while KNN on model internal embeddings indicated with dashed lines. Note that for fine-tuned embeddings, KNN curve is actually strictly higher than the trained classifier.

Classification models are trained to output logit values for each GO class in our dataset, so they cannot be used directly for similarity based comparisons. However, internal model representations, including the embedding before a classification head, may work as representations for K-Nearest Neighbor classifiers (shown in Fig. 3A). We adopt K-Nearest Neighbors to the multi-label setting by estimating the probability of a GO annotation as the inverse distance weighted fraction of neighbors associated to that specific annotation in embedding space. Loss curves on the testing dataset are shown for all models, as well as BLAST for comparison, in Fig. 3C.

Convolutional models optimized for 128 or 2048 dimensions prior to the classifier layer achieve similar performance, both as classifiers and embedding models for KNN. With both representation sizes, KNN annotation has slightly lower performance than direct classification with model logits.

Conversely, for fine-tuned language models, KNNs based on the fine-tuned embedding are more effective than the classifiers themselves, making them a more interpretable classification model with no drawbacks to performance.

To investigate the effect of embedding methods on individual GO classes, we evaluate models on a set of eight GO classes, handpicked to be large but distinctive. Model outputs are arbitrarily thresholded at P=.5 to make discrete classifications, and evaluated by F1 score on testing data, depicted in Fig. 3B. Relying on a KNN improves performance for nearly every sampled class, even when the fine-tuned classifier itself shows poor performance, showing an ability to recover from pathological cases for a direct classifier.

### 3.3 Fine-tuned embeddings generalize to unseen classes

Fine-tuned BERT embeddings are stronger than pre-trained versions for propagating classifications with K-nearest neighbors. However, it is plausible that a fine-tuned model will be over-optimized for its specific target GO classes. We evaluate generalization performance by generating a train/test split not only across proteins, but also across GO classes, so that we can evaluate embedding for KNN performance on classes never previously used to train our model. We then select the BERT-BFD model to be retrained on only one set of GO classes, in order to evaluate the value of fine-tuning towards embedding quality on out of training distribution classes.

**Figure 4.**
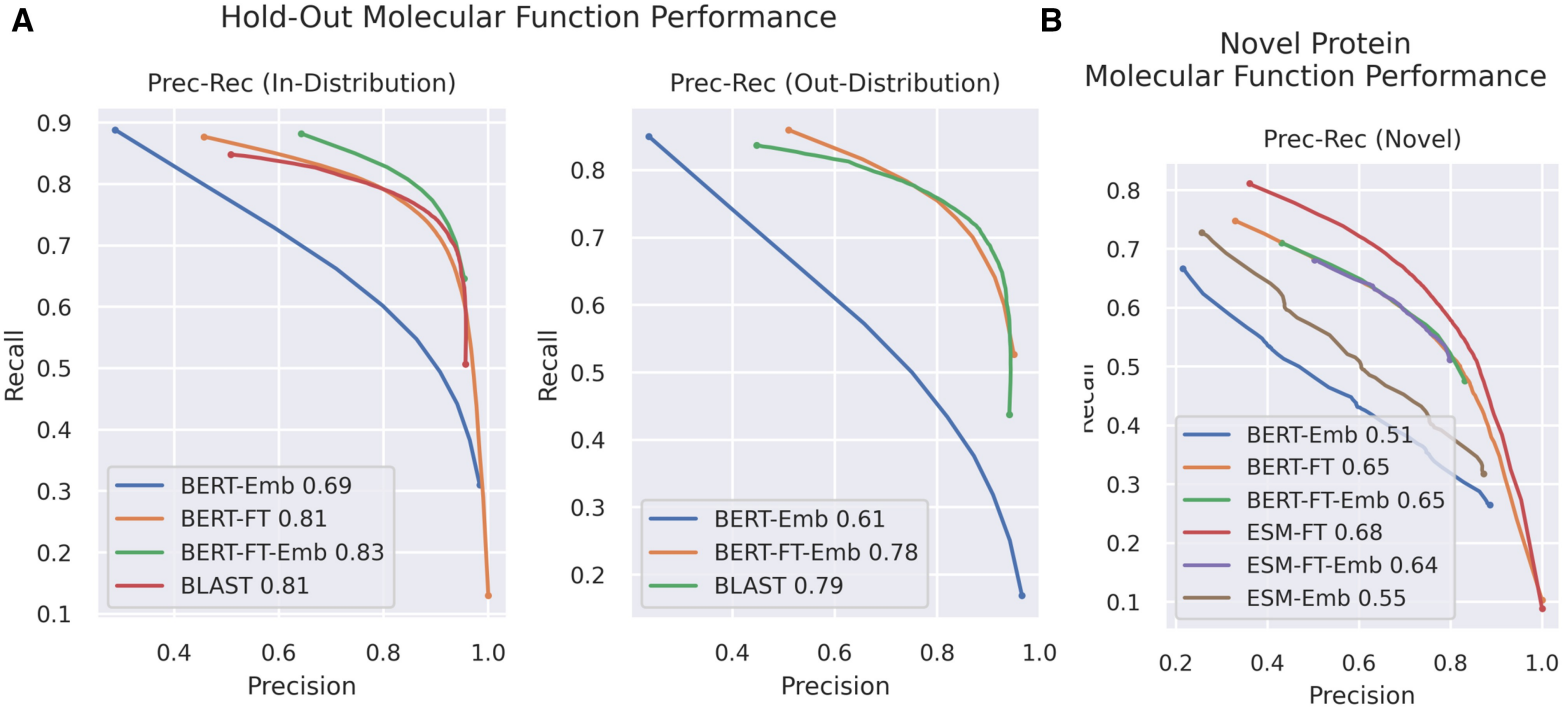
(A) In-distribution performance of selected models on 50% subset of GO terms used for training and out-distribution performance of query based models on 50% subset of GO terms excluded from training. (B) Performance of all models on sequences with no significant BLAST matches in training data. Fine-tuning continues to improve embedding performance for both language models.

As we expect, the relative performances of different models is the same for GO classes included in training data (Fig. 4A). Fine-tuned BERT remains the strongest model, with a KNN based on its internal embeddings still outperforming direct classifications.

For out-of-distribution classes, it is no longer possible to evaluate the classification head, so all referenced models are based on either K-nearest neighbors or BLAST. We evaluate performance curves for each in Fig. 4B. The fine-tuned BERT-BFD model continues to be much stronger than pre-trained embeddings, even for classes not included in training. The gap in F1 score is not directly interpretable, because one set of GO classes may be more difficult to classify than the other, but it is clear that fine-tuned embeddings generalize to novel classes. However, without the benefit of training on known classes, direct sequence comparison with BLAST once again outperforms pure deep learning.

#### 3.3.1 Model performance on novel proteins

Within our testing dataset, most proteins have a handful of high significance (e-value less than 0.001) BLAST hits against the training dataset, which we consider to be strongly related (Altschul *et al.* 1997). Prior models are evaluated on the restricted set of proteins with zero high significance matches against training data. This regime artificially lowers BLAST performance to zero, but simulates a scenario in which a highly novel protein is relevant, such as in a high throughput metagenomic experiment (Pavlopoulos *et al.* 2023), or the study of scarcely characterized proteins. In these scenarios, typical sequence similarity is intractable, so alternative methods are of high interest.

Here, we find that pre-trained embeddings are now slightly stronger than most other models as evaluated by both F-max and S-min (Fig. 4). As expected, all models trained on GO data suffer significantly, but fine-tuned models retain their overall advantage.

### 3.4 Embedding evaluation for unsupervised tasks

Strong document embeddings are generally more effective for unsupervised tasks, such as clustering to discover hidden classes. To evaluate the effects of fine-tuning on general embedding performance, we evaluate embedding performance on a small cross-section of clustering tasks, as well as qualitatively with T-SNE visualizations of embedded GO classes.

We follow the example of the Massive Text Embedding Benchmark (Muennighoff *et al.* 2023), and estimate unsupervised clustering performance as the ability of model embeddings capture existing groups in our datasets. We build several datasets of proteins grouped by similarity into families according to ground truth rules, and evaluate model performance as the v-measure (Rosenberg and Hirschberg 2007) attained by mini-batch k-means run with knowledge of the number of classes and batch size 32.

The HAMAP database (High-quality Automated and Manual Annotation of Proteins) provides a set of 1,983 protein families, selected through complex, manually specified rule sets (Pedruzzi *et al.* 2014). From HAMAP, we randomly select 500 protein families of up to 512 proteins each as a labeled dataset of 216 856 proteins total. No proteins are included in both our GOBench training and HAMAP clustering dataset.

**Figure 5.**
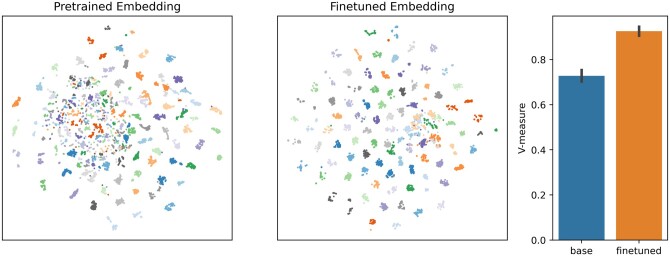
HAMAP Clustering Performance With base BERT language model and GO fine-tuned embeddings. Five hundred protein families of up to 512 proteins each are selected from the HAMAP database and embedded using finetuned and base BERT models. Mini-batch K-means clustering with batch size 32 is run with K=500 to label embeddings. Embedding performance is qualitatively estimated using TSNE visualizations of embeddings, in which clusters are noticeably more clearly defined, and sharper, for the fine-tuned model. Embedding performance is evaluated quantitatively with the sklearn v-measure score of k-means cluster predictions, which ranges from 0 to a maximum score of 1, and calculates a weighted mean of cluster homogeneity and completeness (Rosenberg and Hirschberg, 2007).

Based on qualitative viewing of the t-distributed stochastic neighbor embedding (TSNE) representation of embeddings, as well as the v-measure statistic of clustering quality, we find that fine-tuning significantly increases quality of protein representations for unsupervised discovery of groups. Separate HAMAP families are typically distant within embedding space, with the fine-tuned boundaries sharpened relative to baseline pre-trained results (Fig. 5).

## 4 Discussion

### 4.1 Significance

In this work, we investigate the properties of fine-tuned language models for protein annotation, and we take the novel step of evaluating the impact of fine-tuning on representations for relevant downstream tasks. Language model embeddings are a popular and effective method for annotating proteins (Littmann *et al.* 2021), so simple improvements to their effectiveness has an immediate effect on annotation quality within the field.

We demonstrate that the injection of GO information through fine-tuning immediately improves model for functional classification tasks, and indirectly improves performance in the related area of protein characterization. This is effective not only for interpretable classification, but also for low-resource protein annotation. Nearest neighbor lookup for classification or clustering is highly optimized for both CPU and GPU in packages such as Faiss, and may scale to hundreds of millions of vector lookups (Johnson *et al.* 2021). After precomputation of embedding vectors, KNN classification requires lookup times proportional to the size of the training dataset, like BLAST, but without the need for the high memory GPUs required to run inference with SOTA large language models.

### 4.2 Learning a biologically relevant metric space

Quantitative metrics on classification performance show that the fine-tuning produces a biologically meaningful space of embeddings. Nearby embeddings are likely to share functions, and simply propagating annotations between similar proteins outperforms deep learning alternatives.

**Figure 6.**
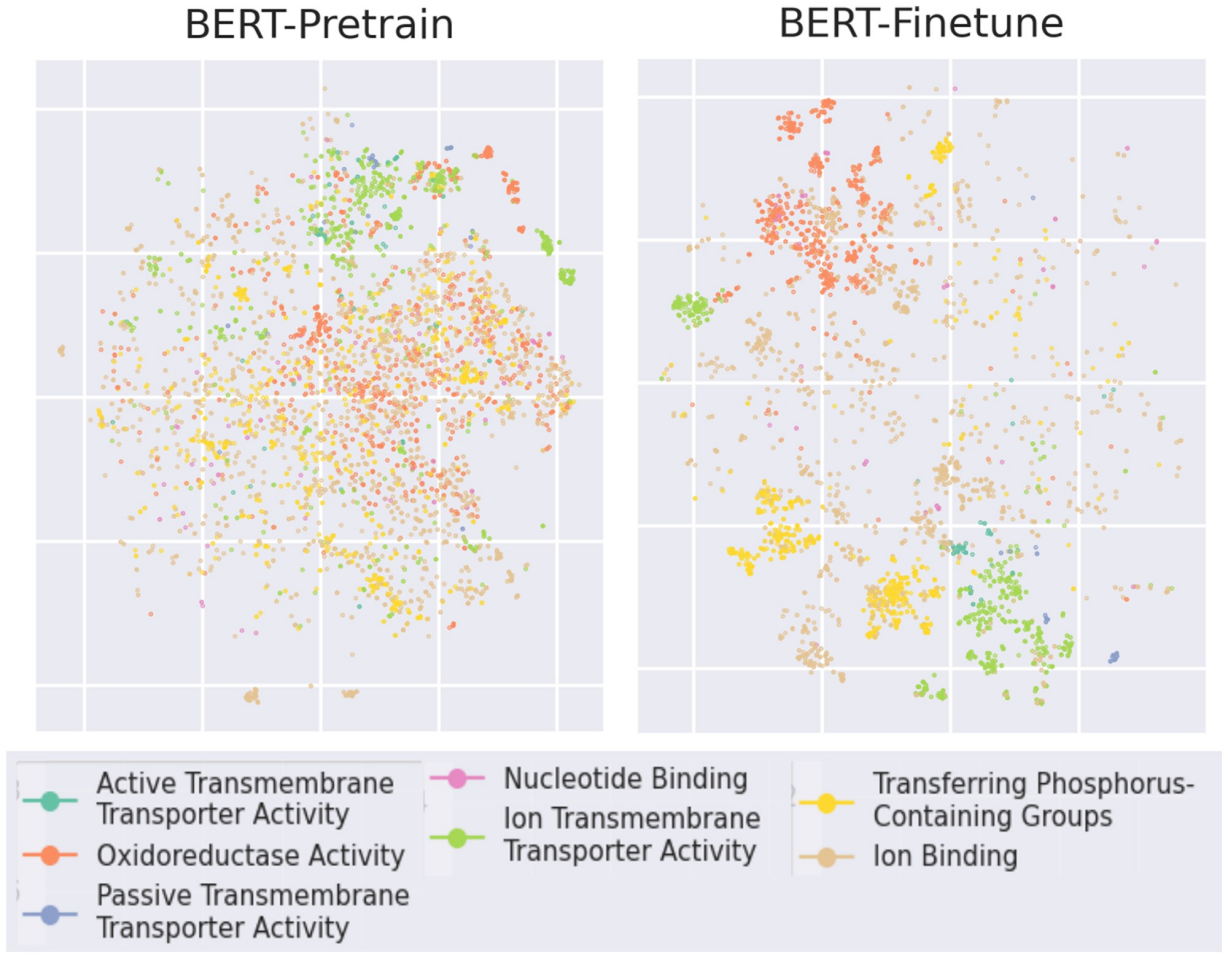
TSNE dimensional reduction of protein embeddings, colored by Gene Ontology classes. Classes are qualitatively selected to be high-level and common, but distinct.

TSNE visualizations of protein representations project them into two dimensions while attempting to maintain significant properties such as pairwise distance between neighbors (van der Maaten and Hinton 2008, Pedregosa *et al.* 2011). TSNE scatterplots of ten significant GO classes indicate that GO classes are somewhat scattered among default pre-trained embeddings, but are indirectly clustered into functionally homogenous groups by fine-tuning classification loss (Fig. 6).

### 4.3 Indirect benefits of fine-tuning

It is not intuitively obvious, or typically true, that K-nearest neighbors on embeddings would outperform machine learning classifiers. Because the gradient descent optimization algorithm is explicitly focused on minimizing classification loss on logits calculated from the embedding, it would be expected that fine-tuning would optimize for this approach specifically. Our convolutional models do show this behavior, and are slightly more effective when classifying with logit predictions. However, LLM embeddings consistently match or outperform logit predictions from the model.

One implication is that there is a significant qualitative difference between pre-trained language model embeddings, and typical embeddings from training on the annotation task exclusively. Additional information from pre-training may make embedding similarity a more useful heuristic, especially in low-data scenarios in the downstream task.

### 4.4 Limitations and extensions of fine-tuning

Fine-tuning a modified architecture improves classification and embedding performance, but is also known to induce forgetting in the pre-trained model (Luo *et al.* 2024), and may actually make embeddings less useful for similarity calculation in low-data scenarios, such as for extremely rare protein classes. More testing is needed to understand the optimal approach to integrating functional data into protein representations. Multimodal models that could handle the conjunction of sequence and functional data simultaneously offer one potential alternative(Brandes *et al.* 2022). At the same time, methods such as continual post-training may help maintain self-supervised knowledge during fine-tuning (Ke *et al.* 2022), and could provide useful alternatives.

In addition, fine-tuning only optimizes embedding lookup performance indirectly, by improving downstream classification. Existing methods for embedding learning, such as contrastive losses, can directly optimize embeddings to correctly segregate distinct object classes (Le-Khac *et al.* 2020). In the future, we hope to explore direct optimization for interpretability through engineered contrastive loss functions.

## Supplementary Material

btae445_Supplementary_Data

## Data Availability

The data underlying this article are available in the GOBench repository at www.gobench.org.
