# Supplementary material for: Fine-tuning protein embeddings for functional similarity evaluation
Source: Bioinformatics. 2024 Jul 10;40(8):btae445. doi: 10.1093/bioinformatics/btae445 (PMC11299545; doi:10.1093/bioinformatics/btae445)
Supplement: btae445_Supplementary_Data [file btae445_supplementary_data.pdf]

Supporting Information for

Fine-tuning Protein Embeddings for Generalizable Annotation Propagation

Andrew M. Dickson and Mohammad R. K. Mofrad (complete author list)

Corresponding Author name.
E-mail: mofrad@berkeley.edu

This PDF file includes:

Supporting text
Figs. S1 to S5
Legends for Dataset S1 to S2
SI References

Other supporting materials for this manuscript include the following:

Datasets S1 to S2

Supporting Information Text

Supplementary Details

Auxiliary Figures. .

.

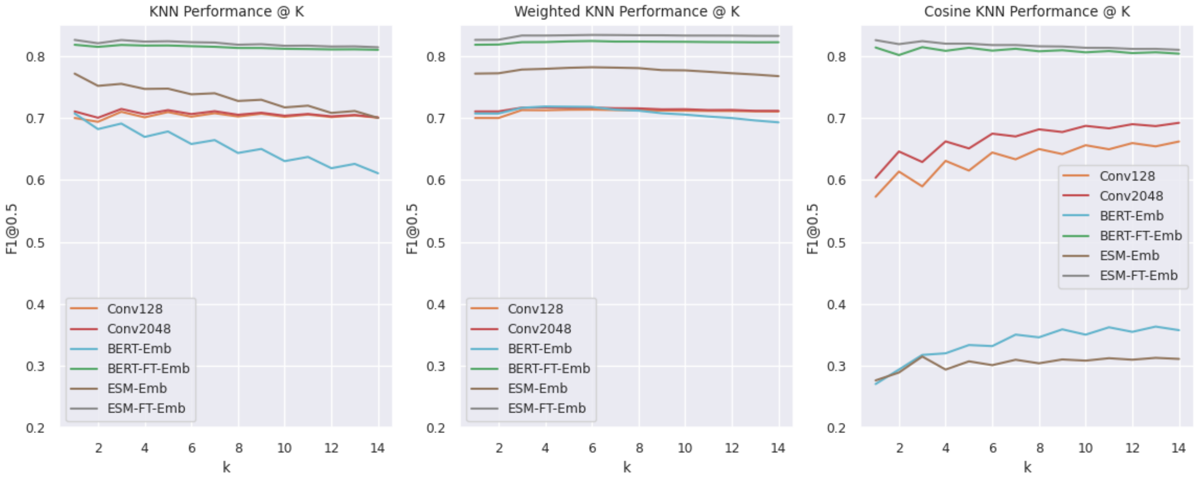

Fig. S1. K-nearest neighbors is for all embedding models for K ranging from 1 to 15. In the default K-nearest neighbors, annotation probabilities for each GO term are estimated as the annotation frequency within the points with the K smallest Euclidean distances. F1 score performance across the Conv128, Conv2048, BERT-Emb, BERT-FT-Emb, ESM-Emb, and ESM-FT-Emb embedding models are measured for values of K ranging from 1 to 15, with performance plateauing around K= for the highest performing embedding models. We also measure KNN performance across K for cosine distance, which achieves similar, but marginally lower, performance for language models, and progressively lower performance for convolutional embeddings. Finally we investigate weighted K-nearest neighbors in which sample importance is inversely scaled with Euclidean distance. As with previous models, inversely weighted KNN plateaus in performances around K=5, but has a marginally higher maximum performance. In addition, inversely weighted KNN is robust to high K values, with no diminishing performance, likely because it correctly discounts unrelated proteins within a neighborhood.

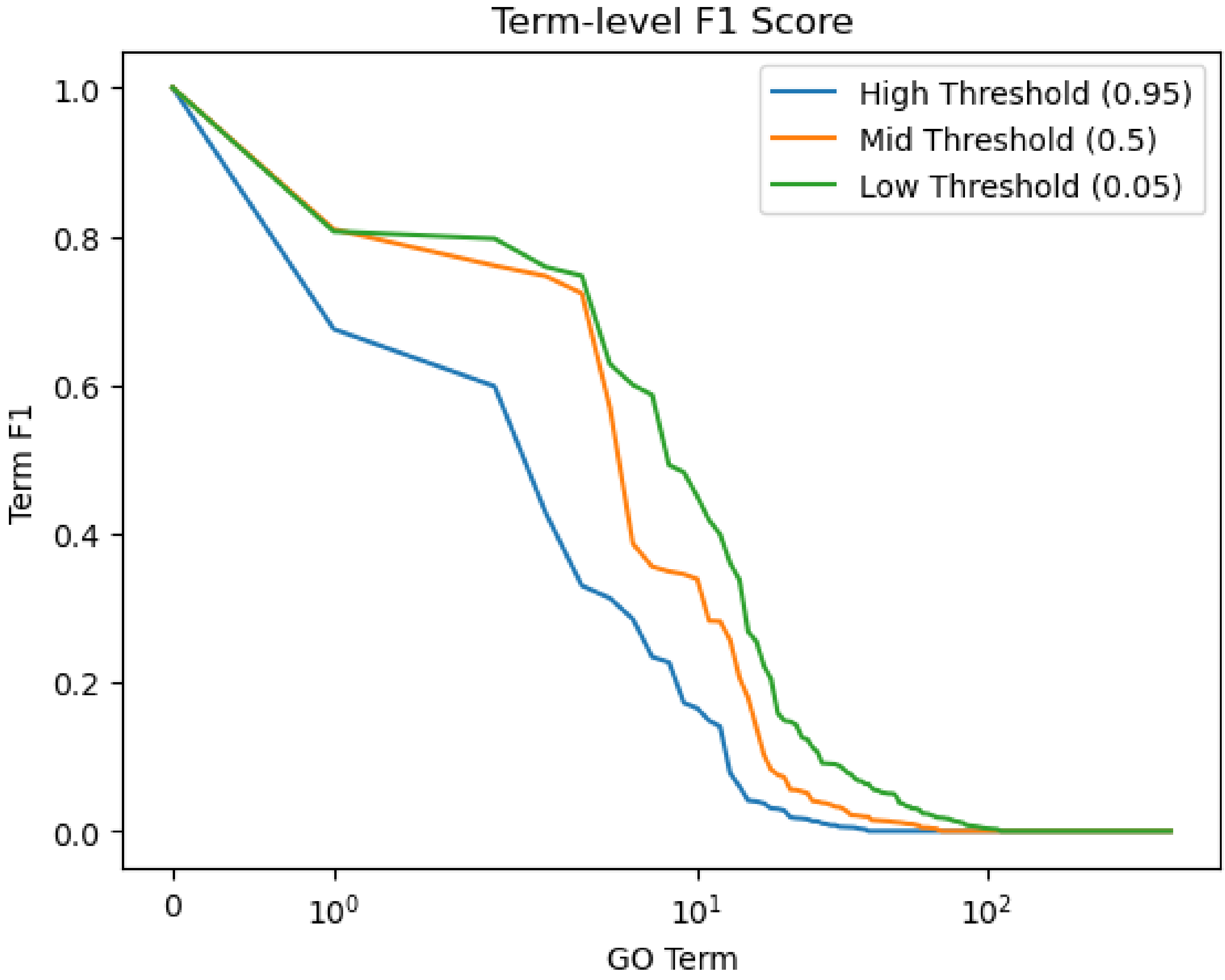

Fig. S2. Relative F1 performance of BERT-CLS model with varying thresholds. Terms sorted by model performance. Model performance appears largely invariant to threshold, with lower thresholds giving somewhat higher final results.

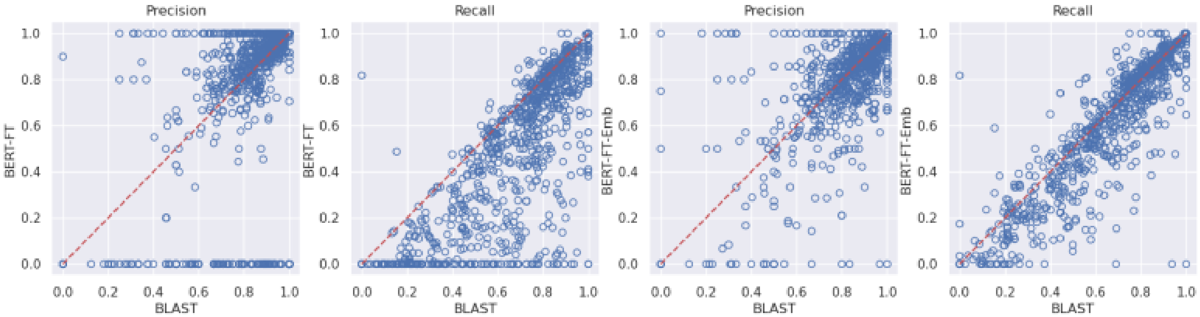

Fig. S3. Scatterplots of precision or recall for each of 865 GO classes included in dataset. X-axis gives precision or recall for baseline BLAST model, while Y-axis shows corresponding value on same GO class for a fine-tuned or embedding model. Points below red line have higher precision or recall when annotated by BLAST.

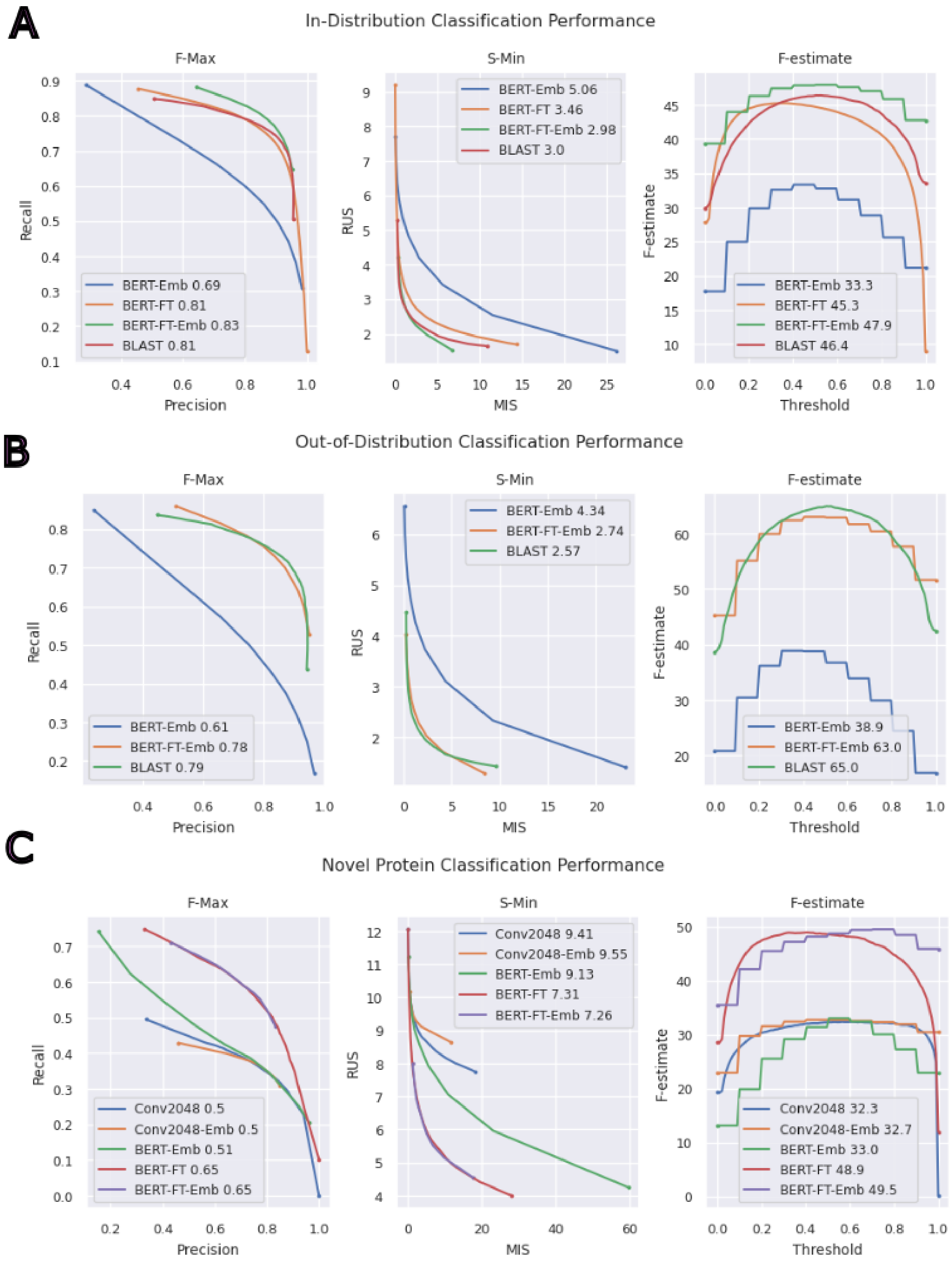

Fig. S4. (A) Performance of selected models on 50% subset of GO terms used for training. (B) Performance of query based models on 50% subset of GO terms excluded from training. (C) Performance of all models on sequences with no significant BLAST matches in training data.

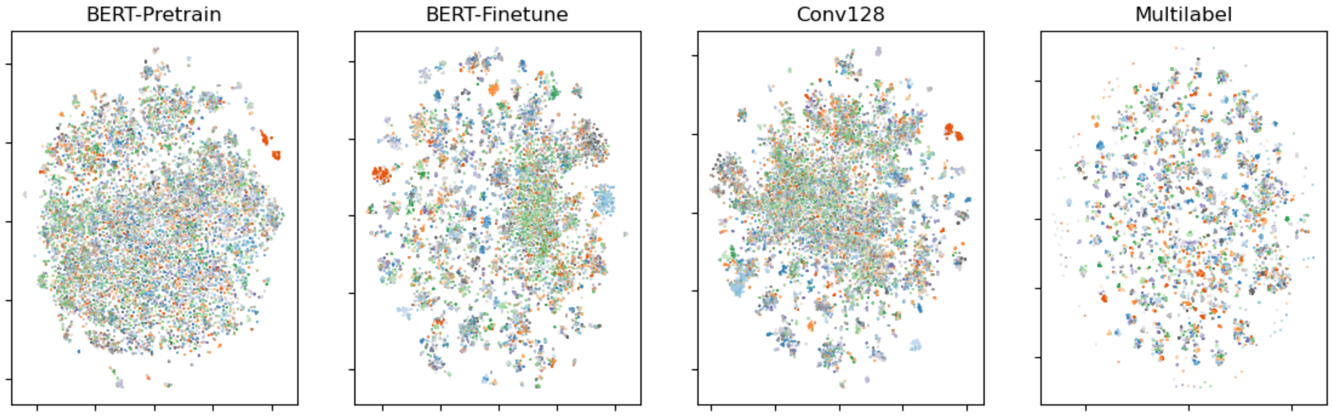

Fig. S5. TSNE-embedding of all testing proteins, colored by an arbitrary similarity metric with similar colors for proteins with similar functions.

Materials. Machine learning training executed on NVIDIA RTX A6000 GPU, using pytorch and pytorch-lightning frameworks. Experiments run through python scripts on lab servers. All code included in github.com/amdson/go_metric repository.

Methods.

.1. F-Max. The classical F1 score is defined for discrete classification predictions on discrete labels, while model outputs are continuous logit values, so before evaluation GO predictions are thresholded by an arbitrary value α. Logit values above α are set as positive predictions, while those below are set as negative. For a given α, define precision as the number of true positive predictions divided by the number of positive predictions and recall as the number of true positive predictions divided by the number positive annotations, with summations over all class labels.

$$pr(\alpha) = \frac{\sum_{i=1}^{N} 1(y_i = \hat{y}_i(\alpha))}{\sum_{i=1}^{N} \hat{y}_i(\alpha)} \quad [1] \qquad rc(\alpha) = \frac{\sum_{i=1}^{N} 1(y_i = \hat{y}_i(\alpha))}{\sum_{i=1}^{N} y_i} \quad [2]$$

The F1 score is then defined as $\frac{2pr(\alpha)rc(\alpha)}{pr(\alpha)+rc(\alpha)}$, and the F-max score is $\max_{\alpha\in[0,1]} F1(\alpha)$. When calculating the F1 score, we use the 'micro-F1' score defined in the python sklearn package (1), and evaluate results over threshold values ranging from 0 to 1 over increments of 0.01 to take the maximum.

.2. S-min. Information content is a quantitative measure of the descriptive value of a gene ontology term, defined using the GO graph structure (2). It associates an importance weighting $ic(g)$ to each GO term $g \in G_n$, representing the amount of information carried by a positive or negative annotation to the term.

With information content, define remaining uncertainty and missing information as the following.

$$ru(\alpha) = \frac{1}{|P|} \sum_{i=1}^{N} ic(g_i) \cdot 1(\hat{y}_i(\alpha) = 0 \wedge y_i = 1) \quad [3]$$

$$mi(\alpha) = \frac{1}{|P|} \sum_{i=1}^{N} ic(g_i) \cdot 1(\hat{y}_i(\alpha) = 1 \wedge y_i = 0) \quad [4]$$

The final S-min score is

$$S_{min} = min_\alpha \left(\sqrt{ru(\alpha)^2 + mi(\alpha)^2} \right)$$

.

.3. F-Score. The F-Score metric is defined to approximate the true F1 score with perfect knowledge of datapoint labels (3). It's based on recall $rc(\alpha)$, and the probability of a positive prediction $P(\hat{y}(\alpha) = 1) = \frac{1}{N} \sum_{i=1}^{N} \hat{y}(\alpha)$. For a given α, the F-Score is

$$\frac{rc(\alpha)^2}{P(\hat{y}(\alpha) = 1)}$$

As with F-max, we take the maximum over all thresholds α for our results.

Baseline Deep Convolutional Model. Convolutional model is a pytorch implementation of the multi-filter model implemented in original DeepGOPlus paper (4). The final model is built out of three main components, and takes proteins as represented by residue sequences of any length as input.

Before processing, a one-hot encoder converts the 22 main protein residue types into binary vector representations, so that protein sequences are input as (L x 22) binary matrices.

Input sequence matrices are first processed by a series of 1D convolutional operators, with a kernels ranging from 3 to 129 in size, and with 800 filters for each kernel. Convolutional operators are initialized with weights from a Xavier distribution and zero bias. Each convolutional operator independently produces an (L x 800) product of the input, which is reduced by pool-max to an 800-dim vector with size independent of sequence length. Vector outputs from each convolutional operator are concatenated to produce a 10,000-dim representation.

The high-dimensional initial vector representation is passed through a two or more multilayer perception layers with ReLU non-linearity before the final output of logit probabilities for each of 865 Gene Ontology classes. Dropout of 0.5 is applied to each layer, with the first bringing the initial size down to only 128 or 2048 dimensions. For all models used in benchmarking, only two MLP layers are used, with the first output being treated as an internal representation for downstream K-nearest neighbor models.

Fine-tuned Large Language Models. For our experiments, we use the ProtBERT-BFD language model pre-trained by RostLab, and the ESM2 650M parameter version. Model weights are available through the Huggingface API, and can be used directly as a Pytorch model.

Tokenizer. Proteins are originally stored as character strings in the FASTA format. We tokenize them into integer arrays using the ProtBERT and ESM2 tokenizers. There is a one-to-one correspondence between the twenty basic amino acids and tokens, with rare additional amino acids being converted to a default 'unknown' index. <cls> tokens are prepended to the sequence, and <pad> tokens are added to the end as needed.

Due to computational constraints, protein sequences are limited to a max length of 1000, and truncated at longer lengths, deviating from the original RostLab methodology for long sequences. When reproducing sequence embeddings from the pre-trained, but not fine-tuned ProtBERT model, proteins are not truncated, and are padded to max length at any size. Batches of shorter sequences are padded to max length.

Training. We follow typical fine-tuning strategies for LLMs, but with the modified multi-label binary classification head. We use the maximum batch size capable of fitting on a single GPU, which is 8 for a sequence length of 1024. Over the course of 7000 training steps, or roughly 1.5 epochs, learning rate is linearly increased from zero to a maximum of 3e-5 for the classifier head, and linearly increased from zero to a maximum of 5e-6 for the original transformer weights. Because of computational constraints, hyperparameters are not methodically hyperparameter tuned, but are set to default values from previous experiments (5).

We find that validation set performance increases continuously over the course of training, and train until we observe diminishing returns at ten epochs.

A. Precision-Recall Across GO Terms. It remains difficult to explain the improved performance of embedding KNN classification over direct usage of models, but plots of precision and recall over GO classes give some hints. When we compare fine-tuned BERT with fine-tuned BERT embeddings, precision is similar across most classes, but recall is significantly improved by the usage of embeddings (Figure S3).

It appears that for many classes, the deep learning classifier almost completely fails to learn annotations for the majority of proteins, but that the similarity heuristic remains useful in these cases, significantly improving recall. Explicit inclusion of this heuristic in future models might close the gap, or surpass KNN methods. At the same time, the high performance of embedding or sequence similarity based heuristics suggests that current deep learning models don't have a significant advantage over interpolation, and may not extrapolate well to new functions.

SI Dataset S1 (gobench.tar.gz)

Gene ontology benchmarking dataset developed using gobench.org. Dataset contains files of form {namespace}_terms.json listing all GO terms included in data, and of form {training/validation/testing}_{namespace}_annotations.tsv listing associated GO terms for each protein ID included in dataset. 'molecular_function' namespace used for paper results. Full description included in GO Bench paper (6).

SI Dataset S2 (uniprot_reviewed.fasta)

FASTA file containing sequences for each protein.

References

1. F Pedregosa, G Varoquaux, et. al., Scikit-learn: Machine learning in Python. *J. Mach. Learn. Res.* **12**, 2825–2830 (2011).
2. WT Clark, P Radivojac, Information-theoretic evaluation of predicted ontological annotations. *Bioinformatics* **29**, i53–i61 (2013).
3. J Bekker, J Davis, Learning from positive and unlabeled data: a survey. *Mach. Learn.* **109**, 719–760 (2020).
4. M Kulmanov, R Hoehndorf, DeepGOPlus: improved protein function prediction from sequence. *Bioinformatics* **36**, 422–429 (2019).
5. A Elnaggar, et al., Prottrans: Toward understanding the language of life through self-supervised learning. *IEEE Transactions on Pattern Analysis Mach. Intell.* **44**, 7112–7127 (2022).
6. A Dickson, E Asgari, AC McHardy, MRK Mofrad, GO Bench: shared hub for universal benchmarking of machine learning-based protein functional annotations. *Bioinformatics* **39** (2023) btad081.
